# Clinical and ultrasonographic features of 104 knee joints in hemodialysis patients: impact of age, gender, and hemodialysis duration: a descriptive cross-sectional study

**DOI:** 10.1186/s12891-025-08447-9

**Published:** 2025-03-10

**Authors:** Samar Tharwat, Marwa Saleh, Rabab Elrefaey, Mona Kamal Nassar, Mohammed Kamal Nassar

**Affiliations:** 1 Rheumatology & Immunology Unit, Department of Internal Medicine, Faculty of Medicine, Mansoura University, Mansoura, Egypt; 2 Department of Internal Medicine, Faculty of Medicine, Horus University, New Damietta, Egypt; 3 Mansoura Nephrology & Dialysis Unit (MNDU), Department of Internal Medicine, Faculty of Medicine, Mansoura University, Mansoura, Egypt; 4 Department of Radiology, Student Hospital, Mansoura University, Mansoura, Egypt; 5 Mansoura University Hospital, El Gomhouria St, Mansoura, Dakahlia Governorate Egypt

**Keywords:** Musculoskeletal ultrasound, Knee, Hemodialysis, Knee effusion

## Abstract

**Background and objectives:**

Hemodialysis (HD) patients struggle with musculoskeletal disorders. This study aimed to examine knee clinical and musculoskeletal ultrasonographic (MSUS) characteristics in HD patients and to evaluate the influence of age, gender, and HD duration.

**Materials and methods:**

This cross-sectional descriptive study included 52 patients (104 knee joints) on regular HD for at least 6 months. Demographic, clinical, therapeutic, and laboratory data were collected. Chronic knee pain was assessed for duration, intensity, and laterality. Swelling, crepitus, and tenderness were assessed in both knees. EULAR-standardized knee MSUS evaluations were performed on all patients. The patients were then compared based on age, gender, and HD duration.

**Results:**

The mean age of the patients was 52.4 ± 14.15 years, with 25 females and 27 males, median duration of HD was 3.5 years. Chronic knee pain was present in 31 of 104 knees (29.8%). The scanned 104 knee joints had at least one MSUS finding in 91 (87.5%): suprapatellar effusion in 57 (54.8%), synovial thickening in 13 (12.5%), abnormal cartilage morphology in 68 (65.4%), quadriceps tendon abnormalities in 58 (55.8%), patellar tendon abnormalities in 34 (32.7), medial meniscus abnormalities in 30 (28.8%), lateral meniscus abnormalities in 13 (12.5%), and Baker cyst in 5 (4.8%). MSUS abnormalities were significantly more prevalent in HD patients older than 40 years (94.9% vs. 65.4; *p* < 0.001). Chronic Knee Pain was more prevalent in females than males (40% versus 20%, respectively). Regarding HD duration, quadriceps, and patellar tendons exhibited more MSUS abnormalities in patients with HD duration > 3 years (86.5% vs. 42.0% and 38.9% vs. 25%, respectively) compared to others.

**Conclusion:**

MSUS findings of the knee are prevalent among HD patients, particularly those of female gender, older age, and longer duration of HD. These findings could be subclinical. Typically, the severity of suprapatellar effusion is related to additional structural abnormalities.

## Introduction

The prevalence and incidence of end-stage renal disease (ESRD) have both been on the rise around the world in recent years [1]. Treatment for ESRD is available in the form of renal replacement therapy (RRT), which undoubtedly influences the patients’ health-related quality of life (HRQoL) [2]. The various forms of renal replacement therapy, such as hemodialysis (HD), peritoneal dialysis (PD), and kidney transplantation (KT), each have their own set of benefits and drawbacks [3].

HD accounts for 89% of all treatments administered worldwide for ESRD patients [4]. Long-term HD does not come without complications, despite being a common management option for ESRD [5]. Among these complications are cardiovascular disease, renal anemia, renal bone disease, and dialysis related amyloidosis [6]. In addition, individuals with chronic HD have a significant burden of musculoskeletal symptoms, which have a poor effect on their HRQoL. Knee pain is one of the prevalent musculoskeletal symptoms that has been overlooked among these patients [7].

Despite the fact that magnetic resonance imaging (MRI) is reliable for assessing bone, articular cartilage, and soft tissues, it is costly, time-consuming, and not commonly available for everyday use in low-resource settings [8]. On the other hand, musculoskeletal ultrasound (MSUS) has many advantages, including its noninvasive nature, low cost, dynamic real-time assessment, and easy side-by-side comparison [9]. MSUS evaluation of the knee in HD patients is a valuable diagnostic tool for identifying musculoskeletal abnormalities that are commonly associated with chronic kidney disease (CKD) and dialysis-related complications. These patients often develop conditions such as patellar tendon thickening, synovial effusion, Baker’s cysts, or calcifications due to chronic inflammation, secondary hyperparathyroidism, and altered calcium-phosphate metabolism [10]. CKD and long-term HD are also associated with metabolic and structural changes, which predispose patients to musculoskeletal abnormalities. While knee joint involvement is a common complaint, many of these abnormalities may be subclinical and go undiagnosed without advanced imaging techniques. MSUS offers a non-invasive method to detect these structural changes early, providing valuable insights into disease patterns and progression. To the best of our knowledge, there are just a few data [11] that have been published in the English literature that have reported the structural sonographic features of the knee joint in chronic HD patients. By assessing the influence of age, gender, and HD duration on knee pathology, this study aims to enhance understanding of these factors and improve early detection and management strategies, ultimately helping to mitigate disability and improve outcomes for HD patients.

Thus, the aim of this study was to determine knee clinical and MSUS features in HD patients and evaluate the influence of age, gender, and HD duration.

## Patients and methods

### Study design and settings

This cross-sectional analytical study was conducted at the Nephrology and Dialysis Unit (MNDU), Internal Medicine Department, Mansoura University Faculty of Medicine, Egypt, from May 2019 to April 2020.

### Study participants (inclusion and exclusion criteria)

Patients diagnosed with ESRD on regular HD made up the target population. The inclusion criteria were as the following: (a) age > 18 years old, (b) a diagnosis of ESRD and on regular HD fore more than 6 months, (c) a willingness to participate in the study. Patients with a history of knee joint fracture, infection, malignancy, intra-articular administration of steroids for at least three months prior to evaluation, or a history of autoimmune or rheumatic diseases were excluded from the study at the outset.

### Ethical consideration

This study was conducted in compliance with the Helsinki Declaration’s principles [12]. The Institutional Research Board of the Faculty of Medicine at Mansoura University approved the study protocol (approval registration number: R.21.11.1531). All participants were provided with detailed information about the study, and their written informed consent was obtained.

### Sociodemographic and clinical data

Sociodemographic data were collected from all participants, including age, gender, education level, occupation, and socioeconomic status. In addition, clinical data were obtained from electronic and written medical records, including the duration of HD and associated comorbidities. Therapeutic data, including erythropoietin, calcium, and iron supplementation, antihypertensives, and antidiabetics, were also collected.

### Rheumatological evaluation of the knee

Each patient was assessed by thorough history-taking and a rheumatological evaluation of both knees. Patients were asked about the presence of pain in one or both knees. Those who reported knee pain were asked to determine the severity of pain through a visual analogue scale (VAS), where 0 meant no pain and 10 meant the worst pain.

For rheumatological evaluation of the knee, each patient was asked to lie in bed, and both knees were inspected for any swelling. Then, the knee joint was palpated for any tenderness or swelling. The patella was tapped to see if there was any effusion deep to the patella. Passive and active flexion of the knee was done to demonstrate the full range of movements and determine if there was any crepitus.

### Musculoskeletal ultrasound of the knee

The EDAN U2 ultrasound device (Shenzhen, China), equipped with a linear array transducer that ranged in frequency from 8 to 13.4 MHz, was used to conduct a real-time MSUS scan of the knee joint. The sonographic parameters were adjusted, and the frequency was set at around 13 MHz to obtain the best US images of the knee structures. We considered incorporating variable frequency adjustments tailored to individual patient characteristics to improve diagnostic precision and better adhere to international standards. A rheumatologist with a minimum of eight years of experience in the field of MSUS performed all sonographic examinations. During the evaluation, the operator was blinded to the clinical status of the patients. Knee joint scanning was performed in accordance with a predetermined protocol based on the technical guidelines of the European Society of Musculoskeletal Radiology [13].

The suprapatellar and parapatellar joint recesses were examined for effusion and synovial hypertrophy as part of the knee US protocol. With full knee flexion, the V-shaped trochlea of the femur and the overlaying articular cartilage were inspected on the axial planes for any abnormalities. Additionally, the thickness of the medial and lateral femoral condylar cartilage was assessed. Long- and short-axis planes were used to map the quadriceps and patellar tendons from their cranial origin to their distal insertion. To determine whether a Baker’s cyst was present, the region located medially between the semimembranosus tendon and the medial head of the gastrocnemius was checked.

**Fig. 1:**
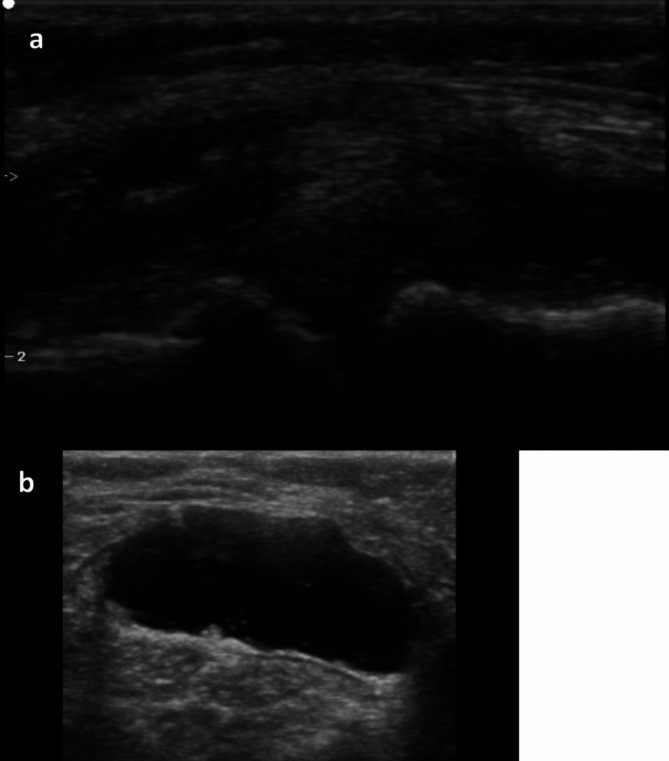
Images of knee US findings in the study HD patients. (**a**) Cartilage abnormality; medial meniscus extrusion. (**b**) Baker cyst

The MSUS images of each patient were collected, and then, using proprietary software, they were reviewed later. Image analysis was performed by one rheumatologist (ST; 8- years’ experience in MSUS) twice with one week interval and one radiologist (MKN; 5-years’ experience in MSUS) who was blinded to the clinical presentation. The US findings were analysed and interpreted using the criteria established by the Outcome Measures in Rheumatoid Arthritis Clinical Trials (OMERACT) US group [14]. Knee effusion and synovial hypertrophy were scored on a semiquantitative scale (where 0 = absence, 1 = mild, 2 = moderate, and 3 = severe) [15]. A disruption of the fibrillar pattern and a hypoechoic appearance of the tendon were used to identify tendon abnormalities. In addition, the quadriceps and patellar entheses were examined for structural abnormalities, bursitis, bone erosions, or calcification that suggests enthesopathy. The individuals were lying supine on the examination bed with their knees bent to their maximal flexion, and measurements of the thickness of the femoral cartilage were taken. US imaging was conducted using the suprapatellar axial view, and measurements were obtained from the midpoints of the medial and lateral femoral cartilage. We evaluated the medial and lateral menisci in longitudinal and transverse planes with the knee positioned in varying degrees of flexion to optimize visualization. Diagnosis of meniscal abnormalities was based on established US criteria, including irregularity, hyperechogenicity, or disruption of the normal triangular morphology of the meniscus. Additionally, the presence of meniscal extrusion beyond the tibial plateau or associated joint effusion was also considered indicative of meniscal pathology. We examined the posterior aspect of the knee medially between the semimembranosus tendon and the medial head of the gastrocnemius to ascertain the presence of a Baker’s cyst. Demonstrative MSUS findings of the knee joint in HD patients are illustrated in Fig. 1.

### Blood sampling and laboratory data

Blood samples were collected from the arteriovenous fistulae (AVF) just before starting the first HD session of the week. Laboratory tests were performed on the days of blood sampling using an automated analyzer. These tests included serum ferritin, transferrin saturation (TSAT), serum calcium (Ca), phosphorus (PO4), parathyroid hormone (PTH), albumin, and a complete blood count.

### Statistical analysis

Data were entered and analyzed using IBM-SPSS software (IBM Corp., released 2019). IBM SPSS Statistics for Windows, Version 26.0. Armonk, NY: IBM Corp.). Qualitative data were expressed as absolute frequency (N) and relative frequency (%, percentage). Quantitative data were initially tested for normality using Shapiro-Wilk’s test with data being normally distributed if *p* > 0.050. Quantitative data were expressed as mean ± Standard deviation (SD) if normally distributed without significant outliers, or median (minimum-maximum) if not normally distributed and/or with significant outliers. The Chi-square test was used to test the association between two nominal variables when the expected count in all cells was ≥ 5, otherwise, Fisher’s exact test was used. The one-way analysis of variance (ANOVA) was used to compare the means of three or more independent groups. The kappa statistic (K) was done to estimate the intra-reader and inter-reader agreement for knee US abnormal findings. The K values were interpreted as follows: k values between 0.00 and 0.20 represented poor; k values between 0.21 and 0.40 represented fair; k values between 0.41 and 0.60 represented moderate; k values between 0.61 and 0.80 represented good; k values between 0.81 and 1.00 represented excellent. Results were considered as statistically significant if *p* value ≤ 0.050.

## Results

Fifty-two patients with ESRD on chronic HD were included in this study, with a mean age of 52.4 ± 14.15 years. There were 25 (48.1%) females and 27 (51.9%) males. Most of them were educated, with about half of them (46.2%) obtaining a high school certificate. Additionally, 53.8% were not employed, and 26.9% had an active lifestyle. The vast majority (76.9%) were non-smokers, and only one patient was an active smoker. The median duration of HD was 3.5 years. Comorbidities included hypertension (71.2%), diabetes mellitus (13.5%), and ischemic heart disease (11.5%). Other clinical, therapeutic and laboratory data were summarized in Table 1.

**Table 1:** Demographic, clinical, and laboratory data of the study HD patients (*n* = 52)

Parameter *n* (%), mean ± SD, median (min-max) (IQR)	HD patients (*n* = 52)
***Demographic data***	
Age	52.4 ± 14.15
Gender	
Female Male	25 (48.1) 27 (51.9)
*Education*	
Not educated Low School Middle School High School College degree Post-graduate	13 (25) 2 (3.8) 4 (7.7) 24 (46.2) 8 (15.4) 1 (1.9)
*Occupation*	
Not employed Employed Retired Not able to work due to disability	28 (53.8) 8 (15.4) 6 (11.5) 10 (19.2)
Active lifestyle	14 (26.9)
*Socioeconomic status*	
Low Average High	23 (44.2) 26 (50) 3 (5.8)
*Smoking*	
Nonsmoker Former smoker Current smoker	40 (76.9) 11 (21.2) 1 (1.9)
Weight, Kg	81.81 ± 16.79
Height, m	1.67 ± 0.08
BMI, Kg/m^2^	29.30 ± 6.27
***Clinical and therapeutic data***	
Duration of hemodialysis, years	3.5 (0.6–13) (3.38)
*Associated comorbidities*	
Diabetes mellitus Hypertension Chronic pulmonary disease Ischemic heart disease	7 (13.5) 37 (71.2) 1 (1.9) 6 (11.5)
*Medications*	
Erythropoietin Calcium supplementation Iron supplementation Antihypertensives Antidiabetics	45 (86.5) 36 (69.2) 28 (53.8) 29 (55.8) 6 (11.5)
***Laboratory data***	
Ferritin, ng/ml	303.95 (12.6–1730) (353.40)
TSAT, %	21 (5–56) (11)
Ca, mg/dL	8.32 ± 0.89
PO4, mg/dL	5.65 (1.9–12.7) (2.65)
PTH, pg./mL	521.5 (9.30–2846) (603)
Albumin, g/dL	3.99 ± 0.30
Hemoglobin, gm/dL	11.13 ± 1.37

The clinical data and MSUS findings of the 104 scanned knee joints are illustrated in Table 2. Of the 104 knee joints, chronic knee pain was reported in 31 (29.8%), with a median duration of 24 months and a median VAS of 7. MSUS findings were detected in the great majority of the knee joints that were examined (87.5%). On clinical evaluation, joint swelling was present in 9 (8.7%), crepitus in 13 (12.5%), and tenderness in 12 (11.5%). Regarding the MSUS findings of the 104 scanned knee joints, knee effusion was detected in 57 (54.8%), while synovial thickening was present in 13 (13.5%). Most of the scanned knees had abnormalities in both cartilage morphology (65.4%) and the quadriceps tendon (55.8%). Baker’s cyst was detected in only five knees. The clinical and MSUS findings of the scanned knees were compared between those below and above 40 years old. Knee pain duration was statistically significantly longer in those over 40 years old (*p* = 0.007). Also, crepitus was significantly detected in those over 40 years old (*p* = 0.026). Regarding MSUS, the presence of knee effusion and its degree of severity were statistically significantly higher in the knee joints of those > 40 years old (*p* = 0.013). Even though, almost all knee joint findings were statistically significantly higher in those above 40 years old, as shown in Table 2.

**Table 2:** Clinical and US findings at the knee joints according to the age in HD patients

Parameter *n* (%), mean ± SD, median (min-max)	All *n* = 52 (Knees = 104)	Age ≤ 40 years *n* = 13 (knees = 26)	Age > 40 years *n* = 39 (knees = 78)	*P*
*Knee Clinical findings*				
Pain	31 (29.8)	10 (38.5)	21 (26.9)	0.265
Joint swelling	9 (8.7)	0	9 (11.5)	0.108
Crepitus	13 (12.5)	0	13 (16.7)	**0.026**
Tenderness	12 (11.5)	2 (7.7)	10 (12.8)	0.726
*Knee ultrasonographic findings*				
Effusion				
Absent Mild Moderate Sever	47 (45.2) 39 (37.5) 15 (14.4) 3 (2.9)	18 (69.2) 8 (30.8) 0 0	29 (37.2) 31 (39.7) 15 (19.2) 3 (3.8)	**0.013**
Synovial thickening				
Absent Mild Moderate Sever	91 (87.5) 6 (5.8) 5 (4.8) 2 (1.9)	*25* (96.2) *0* *1* (3.8) *0*	*66* (84.6) *6* (7.7) *4* (5.1) *2* (2.6)	0.387
Abnormal cartilage morphology	*68* (*65.4*)	8 (30.8)	60 (76.9)	**< 0.001**
Quadriceps tendon abnormalities	*58* (*55.8*)	9 (34.6)	49 (62.8)	**0.012**
Patellar tendon abnormalities	*34 (32.7*)	3 (11.5)	31 (39.7)	**0.008**
Medial meniscus abnormalities	*30 (28.8*)	7 (26.9)	23 (29.5)	0.803
Lateral meniscus abnormalities	*13 (12.5)*	*0*	*13* (16.7)	***0.035***
Baker cyst	*5 (4.8*)	0	5 (6.4)	0.328
Medial femoral cartilage thickness, mm	*2.26 ± 0.75*	2.67 ± 0.60	2.12 ± 0.74	***0.002***
Lateral femoral cartilage thickness, mm	*2.3 ± 0.76*	2.67 ± 0.64	2.18 ± 0.76	***0.005***
US findings	91 (87.5)	17 (65.4)	74 (94.9)	***< 0.001***
One	18 (17.3)	8 (30.8)	10 (12.8)	***< 0.001***
Two	21 (20.2)	2 (7.7)	19 (24.4)	
Three	20 (19.2)	4 (15.4)	16 (20.5)	
More than three	32 (30.8)	3 (11.5)	29 (37.2)	
*HD patients with knee pain*	*31*	*Knees = 10*	*Knees = 21*	
Duration of pain, months	24 (1-168)	*6 (1–60)*	*24 (1-168)*	***0.007***
VAS for pain	*7 (2–10)*	*7 (2–9)*	*7 (2–10)*	*0.898*

Bold values: *p* < 0.05

**Fig. 2:**
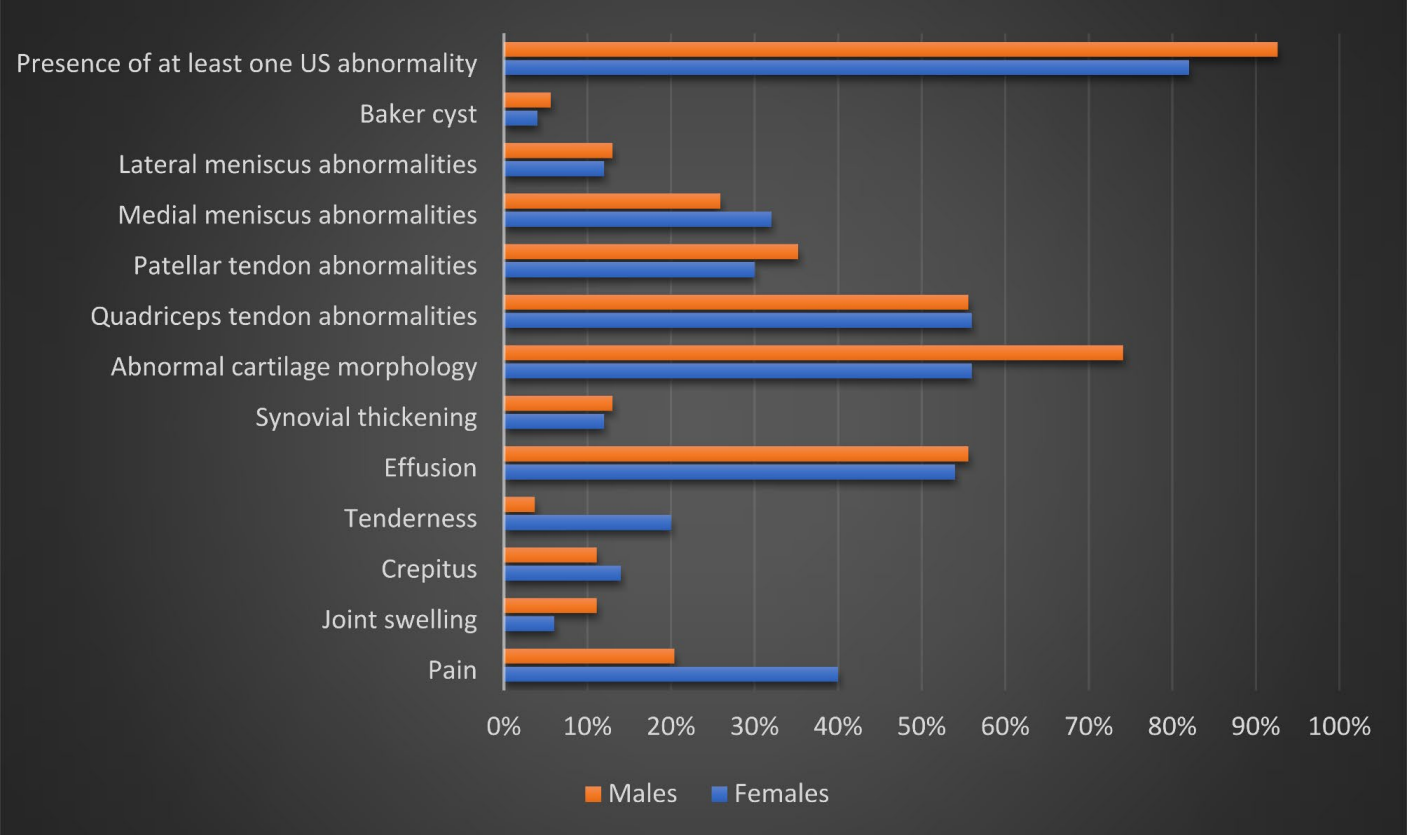
The frequency of clinical and US findings of the knees by gender the study HD patients

Figure 2 shows the difference between both clinical and MSUS knee findings according to gender. Knee pain and tenderness were more prevalent in females while abnormal cartilage morphology was more prevalent in males.

**Fig. 3:**
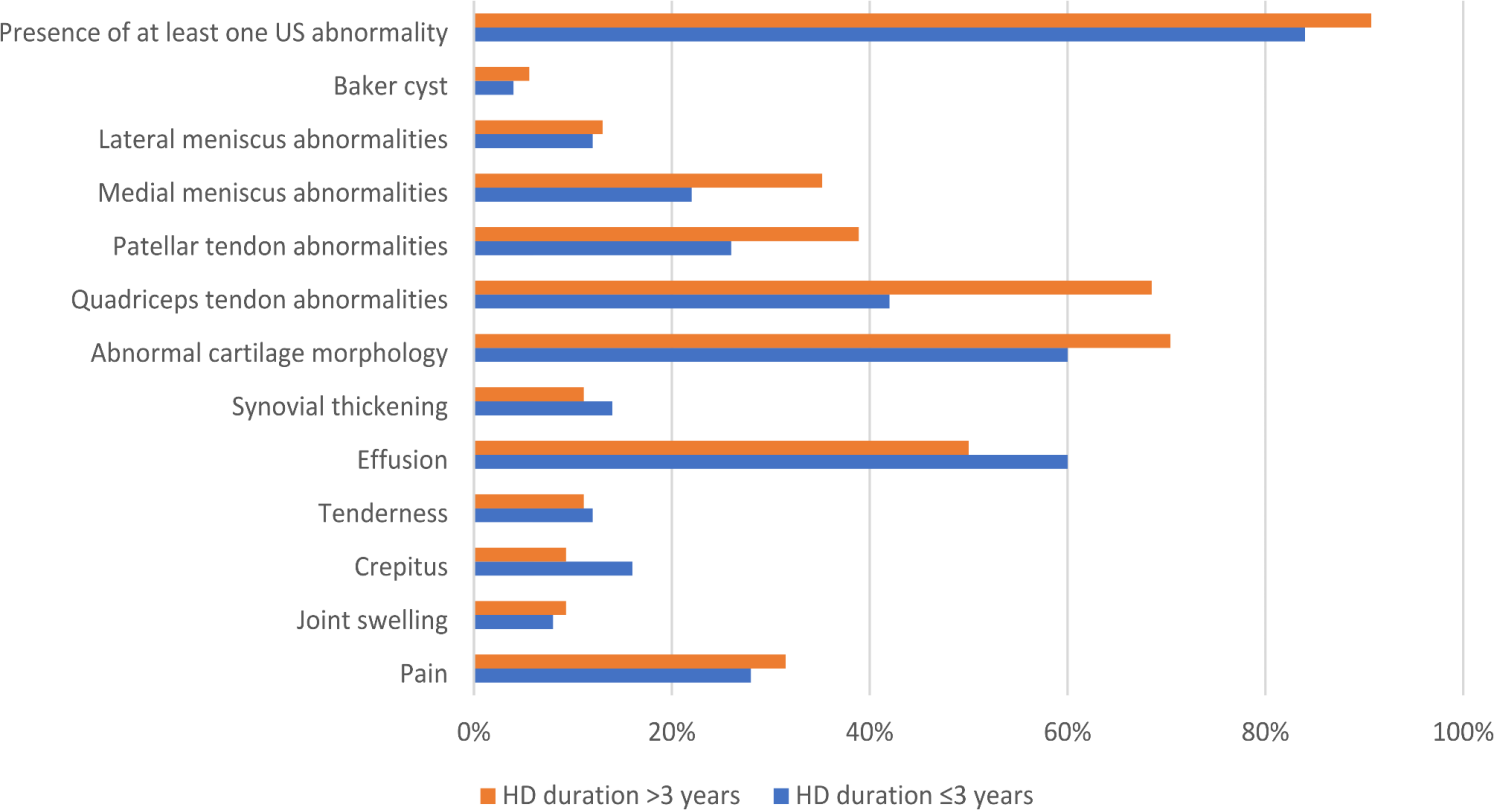
The frequency of clinical and US findings of the knees by duration of HD in the study HD patients

The frequency of clinical and MSUS knee findings was compared according to the HD duration (below or above 3 years). Almost all knee joint findings were more prevalent in those with longer HD durations, while knee effusion was more common in those with HD durations below 3 years, as shown in Fig. 3.

Table 3 shows the prevalence of knee effusion in the scanned knee joints and its relation to the clinical and MSUS findings.

**Table 3:** Prevalence of knee joint effusion and its relation to clinical and US knee findings in the study HD patients

Parameters	Semiquantitative assessment of effusion				*P*
	Absent (Knees = 47)	Mild (Knees = 39)	Moderate (Knees = 15)	Severe (Knees = 3)	
*Knee Clinical findings*					
Pain	11 (23.4)	14 (35.9)	6 (40)	0	0.304
Joint swelling	0	5 (12.8)	2 (13.3)	2 (66.7)	**< 0.001**
Crepitus	2 (4.3)	6 (15.4)	4 (26.7)	1 (33.3)	0.067
Tenderness	1 (2.1)	7 (17.9)	4 (26.7)	0	**0.024**
*Knee ultrasonographic findings*					
Synovial thickening	0	2 (5.1)	8 (53.3)	3 (100)	**< 0.001**
Abnormal cartilage morphology	24 (51.1)	28 (71.8)	13 (86.7)	3 (100)	**0.023**
Quadriceps tendon abnormalities	22 (46.8)	20 (51.3)	13 (86.7)	3 (100)	**0.018**
Patellar tendon abnormalities	9 (19.1)	13 (33.3)	9 (60)	3 (100)	**0.002**
Medial meniscus abnormalities	10 (21.3)	10 (25.6)	7 (46.7)	3 (100)	**0.011**
Lateral meniscus abnormalities	3 (6.4)	6 (15.4)	4 (26.7)	0	0.166
Baker cyst	0	1 (2.6)	1 (6.7)	3 (100)	**< 0.001**
Medial femoral cartilage thickness, mm	2.7 ± 0.74	2.23 ± 0.63	1.64 ± 0.63	1.3 ± 0.15	**< 0.001**
Lateral femoral cartilage thickness, mm	2.7 ± 0.68	2.23 ± 0.76	1.63 ± 0.55	1.1 ± 0.15	**< 0.001**
At least one US finding	34 (72.3)	39 (100)	15 (100)	3 (100)	**< 0.001**

Bold values: *p* < 0.05

Table 4 demonstrates intra- and inter-reader agreement of in detecting knee MSUS findings in the study joints with excellent intra-reader agreement in almost all Knee MSUS findings. The inter-reader agreement varies from good to excellent in knee.

**Table 4:** The intra- and inter-reader agreement in detecting knee ultrasonographic findings in the study joints (*n* = 104)

Knee ultrasonographic findings	The intra-reader agreement				The inter-reader agreement			
	K	SE	*P* value	Strength of agreement	K	SE	*P* value	Strength of agreement
Effusion	0.909	0.035	< 0.001	Excellent	0.895	0.038	< 0.001	Excellent
Synovial thickening	0.882	0.065	< 0.001	Excellent	0.703	0.090	< 0.001	Good
Abnormal cartilage morphology	0.916	0.041	< 0.001	Excellent	0.878	0.048	< 0.001	Excellent
Quadriceps tendon abnormalities	0.922	0.038	< 0.001	Excellent	0.883	0.046	< 0.001	Excellent
Patellar tendon abnormalities	0.956	0.031	< 0.001	Excellent	0.775	0.067	< 0.001	Good
Medial meniscus abnormalities	0.886	0.049	< 0.001	Excellent	0.783	0.069	< 0.001	Good
Lateral meniscus abnormalities	0.753	0.096	< 0.001	Good	0.633	0.105	< 0.001	Good
Baker cyst	0.823	0.122	< 0.001	Excellent	0.695	0.142	< 0.001	Good
Medial femoral cartilage thickness	0.957	0.021	< 0.001	Excellent	0.892	0.032	< 0.001	Excellent
Lateral femoral cartilage thickness	0.968	0.018	< 0.001	Excellent	0.904	0.031	< 0.001	Excellent

## Discussion

To the best of our knowledge, this is the first study to investigate the knee joint findings in chronic HD patients using MSUS and assess the relation of these findings to age, sex, and duration of HD. In this cross-sectional study, we performed clinical and ultrasonographic examinations of a total of 104 knee joints in 52 ESRD individuals who were on chronic HD. Chronic knee pain was evident in nearly one-fourth of the evaluated knees (29.8%), and 91 (87.5%) knee joints had at least one US finding. MSUS findings were more common in females and increased with age and HD duration.

Knee pain is one of the musculoskeletal manifestations that frequently occur in people with chronic HD [7]. Knee pain in chronic HD patients is associated with impaired HRQoL and anxiety. Pain, stiffness, and physical function are worsening with the increased severity of knee pain among these patients. Older age, female gender, a longer duration of HD, and psychiatric disorders are all risk factors that may contribute to this pain [16].

MSUS is a non-invasive, cost-effective, and easily accessible imaging technique that provides real-time visualization of soft tissues, allowing for dynamic evaluation of joint abnormalities. It has been highlighted as an effective tool for detecting early, subclinical changes, monitoring disease progression, and guiding treatment decisions without the need for more complex and expensive imaging modalities like MRI [17]. Given its capacity to assess structures such as tendons, ligaments, cartilage, and synovium, MSUS is particularly beneficial for patients with chronic conditions like those undergoing HD, where early detection and timely intervention are crucial [18].

Even though we detected MSUS findings in the great majority of the knee joints that were examined (87.5%), only 29.8% of those knee joints were symptomatic. Based on these data, a high prevalence of asymptomatic subclinical knee problems in chronic HD patients appears to exist. This is what we found in our prior MSUS studies on chronic HD patients in the evaluation of the shoulder as well as the entheses [19, 20].

In this study, knee effusion was seen in more than half (54.8%) of the scanned knees, but it was mostly mild. In patients with HD, the presence of knee joint effusion is substantially more common than in subjects who have normal renal function [21]. Knee effusion can have a variety of causes, including internal joint derangement or inflammatory arthritis, such as gout or pseudogout, both of which are common among HD patients [22]. Some researchers have shown a link between long-term HD and amyloidosis, which may also be associated with joint effusion [23, 24].

Tendon degeneration is a potential complication of hyperparathyroidism, as well as metabolic acidosis caused by chronic renal failure (CRF) [25–27]. There have been reports in the medical literature of patients with ESRD experiencing spontaneous rupture of their quadriceps or patellar tendons [28–31]. Malta LM and colleagues conducted a cross-sectional, observational, and controlled study to evaluate the knees of individuals with CRF who were on chronic HD, using magnetic resonance imaging (MRI), and compared them with those of a group of individuals with normal renal function. They discovered that patients with chronic HD showed abnormal signals in the quadriceps tendon on MRI [21]. In the current study, MSUS abnormalities of the quadriceps and patellar tendons were found in 55.8% and 32.7% of the scanned knees, respectively. The uremic condition results in the gradual retention of a significant number of substances that are referred to as uremic retention solutes. Previous research has indicated that when these toxins, such as indoxyl sulphate and osteoprotegerin, accumulate in the blood due to renal failure, they are capable of changing bone metabolism and, as a result, potentially collagen metabolism [32]. Also, the accumulation of b,2-microglobulin ( b2-m), causes tendinopathy, and leads to a decrease in the tendon’s flexibility, and makes it more likely to rupture in response to relatively mild force [33]. These mechanisms may eventually be able to explain the pathophysiology and etiology of abnormalities in tendons. In addition, HD causes alterations at the entheseal sites, which get progressively worse with longer periods of HD treatment, particularly after the first ten years of starting HD [34, 35].

Sonoelastography is a relatively new sonographic technique that has lately begun to find application in the study of the musculoskeletal system. The measurement of the deformation response of tissue to an external force or pressure delivered by the ultrasonic transducer is the definition of this term. Depending on the molecular structure, the behavior of each tissue or lesion varies. The response of elastic tissue differs from that of stiffer tissue [36]. Teber MA and colleagues compared sonoelastographic findings for the quadriceps tendon in 53 CRF patients on HD to findings in a control group and discovered that quadriceps tendons in patients with CRF are thinner and have lower elasticity scores than controls [36].

It was not surprising to detect that MSUS findings were much more common in HD patients over the age of 40 compared to others. Jiang and colleagues investigated both knees with MSUS in a community-based cohort study that included 3755 participants, and they discovered that synovial hypertrophy and effusion were increasing with age [37]. Also, osteoarthritis (OA) is a degenerative joint condition that most commonly affects people over the age of 60 [38]. Cartilage degeneration is one of the primary characteristics of this disease [39].

According to the findings of this study, chronic knee pain was shown to be more common in females than in males. This is consistent with the findings of a study by Turkiewicz and colleagues on a total of 10,000 participants to ascertain the frequency of knee pain. The study’s findings showed that the prevalence of frequent knee pain was 25.1% higher in women than in men [40]. In general, women are frequently described as having a higher incidence and prevalence of knee pain [41–43].

In fact, the prevalence of musculoskeletal symptoms is significant in the HD population, and this prevalence rises with increasing years spent receiving dialysis treatment [44–46]. Additionally, problems of the musculoskeletal system tend to cluster according to age and dialysis vintage [10]. In the current study, patients with HD durations of more than three years demonstrated common US abnormalities in their quadriceps and patellar tendons. The occurrence of joint and soft tissue problems may be caused by amyloid deposition, which increases with the duration of HD therapy [47, 48].

In this study, the presence and severity of synovial effusion are linked to other MSUS findings. The existence of effusions in the knee has been proven to have a positive link with internal derangement in the knee by a number of studies [49, 50].

Our study has some limitations, a more representative sample could have increased the validity of our findings. However, there have been very few studies evaluating the parameters defined in our study. Additionally, the inclusion of a healthy control group would enhance the value of the study for the purpose of comparative evaluation. An evaluation of pertinent laboratory data, including uric acid, erythrocyte sedimentation rate, and C-reactive protein, and its correlation with MSUS findings would be highly valuable for the study. To our knowledge, there have been no studies performing a systematic assessment of the morphology of the knee joint in patients with ESRD who are on chronic HD, which makes our study unique. The high prevalence of knee abnormalities detected by MSUS in HD patients underscores the importance of early and comprehensive musculoskeletal evaluations in this population. The findings, including suprapatellar effusion, tendon abnormalities, and cartilage changes, suggest that HD patients are at significant risk for musculoskeletal disorders, which could be subclinical and undiagnosed without proper imaging. Given that chronic knee pain and structural abnormalities were more common in older patients, females, and those with longer durations of HD, these groups may require more targeted monitoring and management to prevent further joint damage and improve their quality of life.

However, future studies should include larger, multicenter cohorts to improve the generalizability of findings and provide a more comprehensive understanding of knee abnormalities in HD patients. Incorporating age- and sex-matched non-HD control groups would allow for direct comparisons, helping to delineate the specific impact of HD on musculoskeletal health. Longitudinal study designs are recommended to explore the progression of knee abnormalities over time and their relationship with HD duration. Investigating the potential effects of therapeutic interventions, such as physical therapy or adjustments in dialysis protocols, on musculoskeletal health in HD patients would also be valuable. Finally, future studies could incorporate advanced imaging modalities, such as MRI, to validate US findings and provide a more detailed evaluation of structural abnormalities.

In conclusion, knee joint MSUS findings are prevalent among HD patients, especially those of female gender, older age, and with a longer HD duration. These findings may be asymptomatic and subclinical.

## Data Availability

The datasets used and/or analysed during the current study are available from the corresponding author on reasonable request.

## Declarations

The manuscript was presented in scientific abstracts at Nephrology Dialysis Transplantation, Volume 37, Issue Supplement_3, May 2022 at the following link: https://academic.oup.com/ndt/article/37/Supplement_3/gfac082.002/6577610.

## Abbreviations

AVF: Arteriovenous fistulae
b2-m: b,2-microglobulin
Ca: Calcium
CRF: Chronic renal failure
CKD: Chronic kidney disease
ESRD: End-stage renal disease
HD: Hemodialysis
HRQoL: Health-related quality of life
KT: Kidney transplantation
MRI: Magnetic resonance imaging
MSUS: Musculoskeletal ultrasound
OA: Osteoarthritis
OMERACT: Outcome Measures in Rheumatoid Arthritis Clinical Trials
PD: Peritoneal dialysis
PO4: Phosphorus
PTH: Parathyroid hormone
TSAT: Transferrin saturation
VAS: Visual analogue scale
